# Preimplantation genetic testing for aneuploidy optimizes reproductive outcomes in recurrent reproductive failure: a systematic review

**DOI:** 10.3389/fmed.2024.1233962

**Published:** 2024-02-07

**Authors:** Youwen Mei, Yonghong Lin, Ying Chen, Jiafeng Zheng, Xue Ke, Xuefei Liang, Fang Wang

**Affiliations:** Department of Reproduction and Infertility, Chengdu Women's and Children's Central Hospital, School of Medicine, University of Electronic Science and Technology of China, Chengdu, China

**Keywords:** preimplantation genetic testing for aneuploid, recurrent reproductive failure, recurrent implantation failure, recurrent pregnancy loss, comprehensive chromosomal screening, blastocyst biopsy, reproductive outcomes

## Abstract

**Introduction:**

Recurrent reproductive failure (RRF) is a common pregnancy complication, imposing great physical, emotional and financial burden for the suffered couples. The leading cause of RRF is believed to be aneuploid embryo, which could be solved by preimplantation genetic testing for aneuploidy (PGT-A) in theory. With molecular genetic development, PGT-A based on comprehensive chromosomal screening (CCS) procedures and blastocyst biopsy is widely applied in clinical practice. However, its effects in RRF were not defined yet.

**Methods:**

A systematic bibliographical search was conducted without temporal limits up to June, 2023. Studies about the effects of PGT-A based on CCS procedures and blastocyst biopsy in RRF were included.

**Results:**

Twenty studies about the effects of PGT-A based on CCS procedures and blastocyst biopsy in RRF were included. It revealed that PGT-A could optimise the reproductive outcomes of RRF sufferers, especially in those with advanced age. However, in patients with multiple occurrences of pregnancy losses, the benefits of PGT-A were limited.

**Discussion:**

More randomized controlled trials with large sample size are required to evaluate the benefits of PGT-A in RRF sufferers and identify which population would benefit the most.

## 1 Introduction

Recurrent reproductive failure (RRF), a common pregnancy complication, mainly comprises recurrent pregnancy loss (RPL) and recurrent implantation failure (RIF) (1). RPL refers to ≥2 pregnancy losses or miscarriages (recurrent miscarriage, RM) before 20–24 weeks of gestation (2), affecting 1–2% of all couples (3), while RIF is defined as ≥3 failed embryo transfers with good-quality in *in vitro fertilization* (IVF) (4), affecting about 10% of couples undergoing IVF treatment (5). RRF had brought great physical and mental pressure to the suffered couples, which linked to increased risk of infertility and pregnancy loss (6).

Aneuploidy is a critical cause of RRF (7). In RPL, aneuploidy is identified in at least 55% of products of RPL sufferers’ conception (8), while the embryo is thought to be responsible for 30–50% of RIF (9). Therefore, euploid embryo transfer (ET) is speculated to optimize the reproductive outcomes of RRF. Fortunately, euploid embryos could be selected by preimplantation genetic testing for aneuploidy (PGT-A). Originally, PGT-A was achieved by fluorescence *in situ* hybridization procedure (10), which was highly limited since it assessed only nine out of 24 chromosomes simultaneously with low resolution (11). Multiple major professional societies recommended against its general use (12), as PGT-A based on the FISH procedure failed to improve reproductive outcomes in clinical practice (13, 14). With molecular genetic advances, comprehensive chromosomal screening (CCS) procedures and blastocyst biopsy were developed in PGT-A. The commonly used CCS procedure encompasses array comparative genomic hybridization (aCGH), quantitative real-time PCR (qRT-PCR) and next-generation sequencing (NGS), etc. (12). CCS procedures not only analyzed the number of all chromosomes, but also segmental abnormalities. As blastocysts could better tolerate the insults of biopsy with an increased accuracy rate, blastocyst biopsy was found to yield overall improved reproductive outcomes than cleaved embryo biopsy or polar biopsy (12, 15, 16). Therefore, PGT-A based on CCS procedure and blastocyst biopsy is widely used in clinical practice. However, no consensus has been reached about its effects in RRF sufferers. The review aims to investigate if PGT-A is beneficial for RRF sufferers, and identify suitable population by sub-group analysis.

## 2 Methods

### 2.1 Search strategy and study selection

Databases of PubMed, Embase, and Cochrane Central Register of Controlled Trials were searched with the following terms: (recurrent OR repeated OR habitual) AND (pregnancy loss OR spontaneous abortion OR miscarriage OR fetal wastage) AND (preimplantation genetic diagnosis OR preimplantation genetic screening OR euploid OR preimplantation genetic test) from inception to June, 2023. The inclusion criteria were as follows. Published in English in peer-reviewed journals; irrespective of study-design; studies focusing on the impact of PGT-A based on CCS procedures and blastocyst biopsy on the reproductive outcomes of RRF sufferers. Commentaries, letters, reviews, conference abstracts, and irrelevant studies were excluded. Studies in which PGT-A assay not based on CCS procedure and blastocyst biopsy were also excluded.

### 2.2 Study screening and data extraction

**Figure 1:**
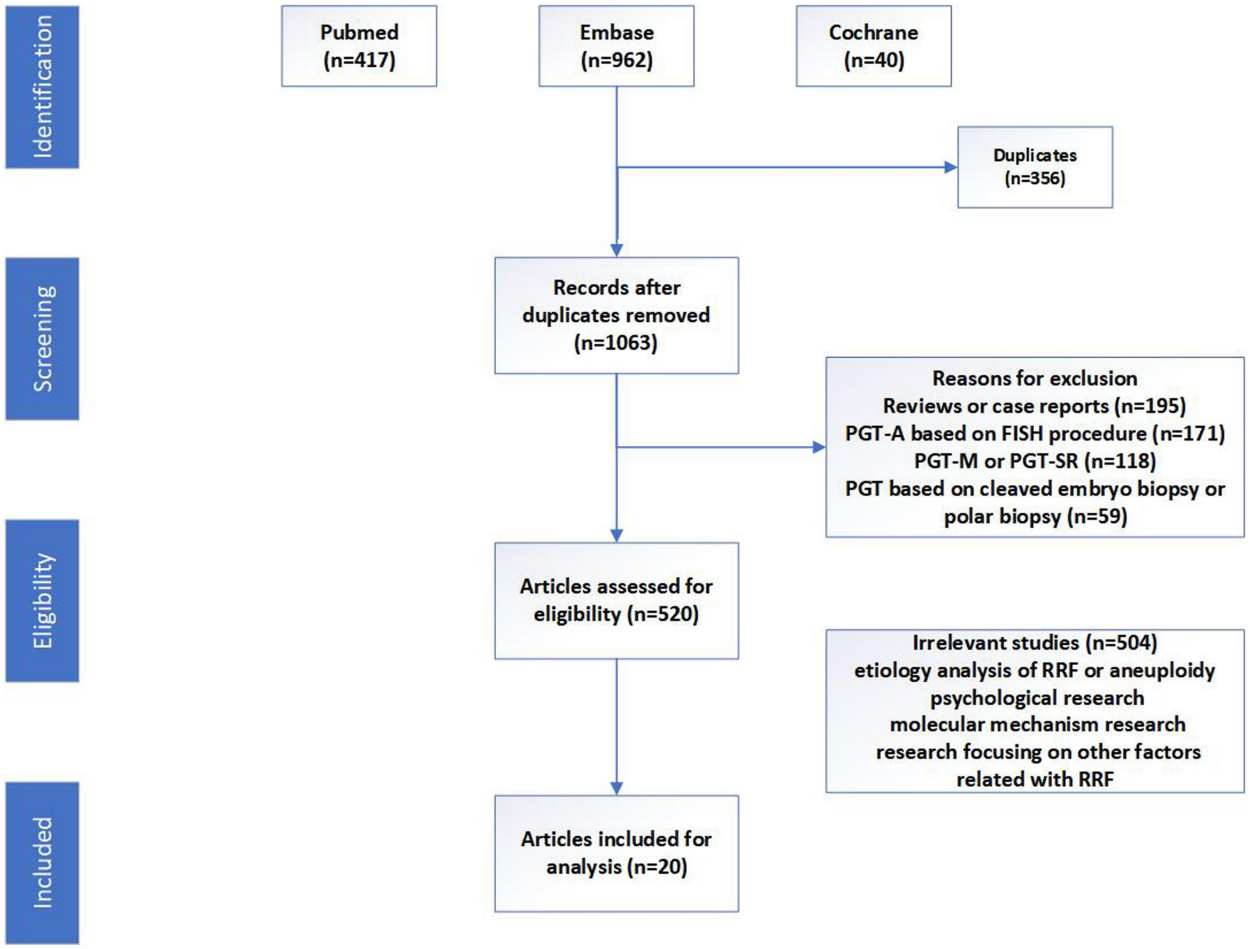
The search strategy diagram.

Following an initial search and duplicates removed, a total of 1,063 literatures were screened by two authors independently (XK and XL). An overview of and screening process is presented in Figure 1. The selected studies were comprehensively examined, and the relevant data were extracted according to our developed data extraction spreadsheet by authors (YM and YL). Information selected included author’s name, publication year and country of the study, study year, study aim, sample size, methodology, sample characteristics, and outcome measures. Any discrepancies would be resolved by discussion until consensus was reached. The primary outcomes of interest were live birth rate (LBR), defined as the percentage of couples achieving a live birth after 24 weeks’ gestation. Secondary outcomes of interest included implantation rate (IR), clinical pregnancy rate (CPR)/ongoing pregnancy rate (OPR), biochemical pregnancy loss (BPL) rate, and miscarriage rate (MR). This study was exempted from Institutional Review Board approval, as it was a systematic review. We adhered to the Preferred Reporting Items for Systematic Reviews and Meta-Analyses (PRISMA) guidelines (17).

## 3 Results

### 3.1 Study characteristics

Unfortunately, only two prospective studies were retrieved. The other 18 included studies were all retrospective. Meta-analysis was precluded owing to their great heterogeneity. Table 1 showed the basic clinical characteristics of the included studies. These studies were all published between 2014 and 2023, and most (*n* = 13) published between 2019 and 2023. The geographical spread of these studies was as follows: seven from China, five from the United States, three from India, two from Italy, and one from Japan, Greece, and Latvia, respectively. In the present review, the study population consisted of RRF sufferers using PGT-A, whereas the control population comprised of RRF sufferers not using PGT-A or those without RRF history using PGT-A. RPL/RIF PGT-A refers to RPL/RIF sufferers who underwent PGT-A. RPL/RIF NO PGT-A refers to RPL/RIF sufferers who did not undergo PGT-A. NO RPL/RIF PGT-A refers to patients without a history of RPL/RIF who underwent PGT-A. The reproductive outcomes were presented in Table 2.

**Table 1:** Basic clinical characteristics of the included studies.

Reference	Country	Study year	Research method	Inclusion criteria	PGT assay	Study group	Number of patients	Age [mean ± standard deviation or median (range)]	BMI [mean ± standard deviation or median (range)]	AMH [mean ± standard deviation or median (range)]
Greco et al. (18)	Italy	2012–2013	Retrospective	RIF, NO RIF, aged <36	CGH	RIF PGT-A	43	32.8 ± 3.1	-	4.1 ± 1.1
						RIF NO PGT-A	33	31.5 ± 2.9	-	5.2 ± 2.4
						NO RIF PGT-A	45	31.7 ± 2.9	-	4.6 ± 1.2
Murugappan et al. (19)	United States	2009–2014	Retrospective	RM	NGS	RM + PGT-A	112	37.1 ± 4.1	23.5 ± 3.0	-
						RM	188	35.7 ± 3.9	24.3 ± 4.8	-
			Subgroup analysis			RM PGT-A: proceeded	-	37.3 ± 3.9	-	-
						RM PGT-A: canceled	-	37.6 ± 4.3	-	-
Sato et al. (20)	Japan	2017–2018	Multicenter prospective	RM (≥ 1 miscarriage caused by aneuploid embryo), RIF	CGH	RM PGT-A	41	39.2 ± 2.05	21.1 ± 2.86	-
						RM	38	39.3 ± 2.07	21.7 ± 2.45	-
						RIF PGT-A	42	38.6 ± 2.06	21.6 ± 2.68	-
						RIF	50	38.7 ± 2.15	21.7 ± 3.07	-
Kim et al. (21)	United States	2012–2018	Retrospective	RPL, infertile patients	NGS	RPL PGT-A	660	-	-	-
						RPL	101	-	-	-
						NO RPL PGT-A	3,975	-	-	-
Lee et al. (22)	Taiwan	2012–2015	Retrospective	RIF, idiopathic RM, OD	CGH	idiopathic RM PGT-A	82	34.8 ± 4.3	-	-
						RIF PGT-A	82	35.8 ± 4.2	-	-
						OD PGT-A	45	24.8 ± 3.0	-	-
Wang et al. (23)	United States	2014–2018	Retrospective	RM	NGS	No miscarriage PGT-A	183	35.7 (3.7)	-	-
						1 miscarriage PGT-A	59	35.5 (mean)	-	-
						RM PGT-A	41	36.1 (mean)	-	-
Sui et al. (24)	China	2020	Prospective randomized	RPL	SNP	RPL PGT-A	103	35.73 ± 4.76	22.00 (20.31–23.43)	-
						RPL	104	35.92 ± 5.10	21.66 (20.51–23.80)	-
Mantravadi et al. (25)	India	2014–2019	Retrospective	Idiopathic RPL	NGS	RPL PGT-A	82	-	-	-
						RPL	30	-	-	-
Ni et al. (26)	China	2013–2018	Retrospective	RIF, BPL, RM, Con (≤ 2IFs, ≤1BPL, ≤1EM, and no LM) aged 20–38	CGH,NGS	Con PGT-A	103	33.00 (30.00–35.00)	22.98 (20.77–25.71)	2.88 (1.65–4.78)
						RIF (3) PGT-A	41	31.00 (29.50–34.00)	22.84 (21.61–25.17)	4.16 (2.30–5.88)
						RIF (4) PGT-A	36	33.00 (30.25–35.00)	22.48 (20.81–24.49)	2.89 (1.33–4.27)
						RIF (≥5) PGT-A	32	34.00 (31.25–35.00)	21.56 (20.47–26.88)	3.69 (1.44–5.57)
						BPL (2-3) PGT-A	51	32.00 (29.00–34.00)	23.59 (21.48–26.97)	3.97 (2.12–6.53)
						BPL(≥4)PGT-A	17	31.00 (30.00–34.00)	23.34 (21.15–26.01)	3.83 (2.15–6.85)
						Early RM (2) PGT-A	177	33.00 (29.00–35.00)	23.03 (21.28–25.71)	3.19 (1.73–5.51)
						Early RM (3) PGT-A	123	32.00 (29.00–34.00)	23.56 (21.80–25.40)	3.62 (1.83–5.88)
						Early RM (4) PGT-A	56	33.00 (30.00–35.00)	23.67 (21.89–26.00)	4.23 (2.15–5.74)
						LM (≥1) + PGT-A	16	33.00 (29.50–35.75)	23.85 (21.01–25.17)	3.21 (2.37–4.89)
Liu et al. (27)	China	2015–2018	Retrospective	Idiopathic RPL, those had PGT-M	SNP, NGS	iRPL aged<35 PGT-A	30	31.8 ± 2.8	21.2 ± 2.8	-
						iRPL aged >35 PGT-A	32	39.0 ± 2.6	23.3 ± 2.9	-
						aged<35 PGT-M	157	30.4 ± 3.1	21.1 ± 2.9	-
						aged>35 PGT-M	57	39.4 ± 2.9	22.0 ± 2.3	-
Mantravadi et al. (25)	India	2014–2019	Retrospective	RPL	NGS	RPL PGT	82	-	-	-
						RPL NO PGT	30	-	-	-
Fodina et al. (28)	Latvia	2017–2020	Retrospective	RIF	aCGH or NGS	RIF PGT	87	36.0 (38.0–34.0)		
						RIF NO PGT	72	34.0 (37.0–32.0)		
Bhatt et al. (29)	The United States	2010–2016	Retrospective	RPL	CGH, NGS	RPL PGT-A	3,351	36.2 ± 4.1	-	-
						RPL	3,241	36.5 ± 4.6	-	-
Lei et al. (30)	China	2014–2017	Retrospective	RPL	NGS	RPL PGT-A	212	35.4 ± 5.0	22.1 ± 2.5	4.35 ± 3.45
						RPL	294	35.0 ± 5.2	22.2 ± 3.0	3.47 ± 3.40
Rao and Mantravadi (31)	India		Retrospective		NGS	RIF PGT-A	54	-	-	-
						RIF	189	-	-	-
Cozzolino et al. (32)	Italy	2013–2018	Retrospective	RIF aged 18–45	NGS	M-RIF >3	1840	37.9 (37.7–38.1)	23.2 (22.8–23.6)	-
						M-RIF > 3 PGT-A	144	38.2 (38.0–38.5)	22.6 (21.9–23.2)	-
						S-RIF > 5	408	38.5 (38.1–39.0)	23.2 (23–23.4)	-
						S-RIF > 5 PGT-A	53	38.3 (38.1–38.6)	22.2 (21.1–23.3)	-
Gu et al. (33)	China	2014–2021	Retrospective	RIF	NGS	RIF PGT-A	209	36.1 ± 3.3	20.89 ± 3.20	3.95 ± 2.87
						RIF NO PGT-A	257	33.4 ± 3.9	20.25 ± 3.07	3.59 ± 2.95
Pantou et al. (34)	Greece	2017–2019	Retrospective	RM, RIF	aCGH	RM PGT	25	35.9 (31–45)		
						RM NO PGT	40	33.5 (28–36)		
						RIF PGT	30	34.7 (29–39)		
						RIF NO PGT	42	33.4 (28–36)		
Pavlovic et al. (35)	The United States	2017–2021	Retrospective	Idiopathic RPL	-	RPL PGT	442	26.6 ± 5.5	-	4.8 ± 4.2
						RPL NO PGT	147	25.2 ± 5.1	-	3.8 ± 3.1
Du et al. (36)	China	2017–2021	Retrospective	RIF	NGS	RIF PGT-A	59	-	-	-
						RIF NO PGT-A	119	-	-	-

“-” refers to the missing data. PGT-A, Preimplantation genetic testing for aneuploidy; RRF, Recurrent reproductive failure; RIF, Recurrent implantation failure; RPL, Recurrent pregnancy loss; RM, Recurrent miscarriage; OD, Oocyte donors; and RM, Repeated miscarriage.

**Table 2:** The comparison of reproductive outcomes between the study group of patients with RRF using PGT-A and the control group.

Reference	Study group	β-HCG+ Rate	IR	CPR	OPR	LBR	BPLR	MR
Greco et al. (18)	RIF PGT-A	82.90%	68.30%	68.30%	-	-	4.90%	-
	RIF NO PGT-A	27.30%	22%	21.20%	-	-	6.10%	-
	NO RIF PGT-A	84.10%	70.50%	70.50%	-	-	9.10%	-
Murugappan et al. (19)	RM PGT-A	-	-	44%	-	32%	-	20%
	RM	-	-	51%	-	34%	-	24%
Sato et al. (20)	RM PGT-A	76.20%	-	66.70%	-	52.4%, 26.8%^a^	12.50%	14.30%
	RM	54.10%	-	29.70%	-	21.6%, 21.1%^a^	45.00%	20.00%
	RIF PGT-A	79.20%	-	70.80%	-	62.5%, 35.7%^a^	10.50%	11.80%
	RIF	53.70%	-	31.70%	-	31.7%, 26.0%^a^	40.90%	-
Kim et al. (21)	RPL PGT-A	-	-	73%	65%	62%	-	15%
	RPL	-	-	61%	55%	41%	-	32%
	NO RPL PGT-A	-	-	72%	67%	63%	-	12%
Lee et al. (22)	RM PGT-A	-	49.10%	63.2%, 52.4%^b^	-	55.9%, 46.3%^b^	-	7.00%
	RIF PGT-A	-	45.70%	57.8%, 45.1%^b^	-	51.6%, 40.2%^b^	-	8.10%
	OD PGT-A	-	52.90%	71.4%, 66.7%^b^	-	57.1%, 53.3%^b^	-	16.70%
Wang et al. (23)	No miscarriage PGT-A	68.90%	-	-	53.60%	-	15.10%	6.3%
	1 miscarriage PGT-A	76.30%	-	-	54.20%	-	20%	8.90%
	RM PGT-A	65.90%	-	-	43.90%	-	22%	11.10%
Sui et al. (24)	RPL PGT-A	-	-	51.3%, 55.34%^a^	49.57%, 55.34%^a^	43.48%, 48.54%^a^	-	1.74%,0^a^
	RPL	-	-	31.41%, 44.23%^a^	19.87%, 29.81%^a^	18.59%, 27.88%^a^	-	11.54%, 14.12%^a^
Mantravadi et al. (25)	RPL PGT-A	-	-	-	-	32.32%	-	9.68%
	RPL	-	-	-	-	30%	-	23.33%
Ni et al. (26)	Con (≤ 2IFs, ≤1BPL, ≤ 1 EM, and no LM) PGT-A	-	58.91%	-	-	53.49%,66.99%^b^	-	6.58
	RIF (3) PGT-A	56.10%	50.91%	-	-	49.09%,65.85%^b^	-	3.57
	RIF (4) PGT-A	-	63.64%	-	-	52.27%,63.89%^b^	-	10.71
	RIF (≥5) PGT-A	-	55.00%	-	-	47.5%,59.38%^b^	-	13.64
	BPL (2-3) PGT-A	55.50%	53.62%	-	48.80%	46.38%,62.75%^b^	-	10.81
	BPL(≥4) PGT-A	-	61.90%	-	-	57.14%,70.59%^b^	-	7.69
	early RM (2) PGT-A	60%	62.08%	-	48.10%	53.02%,69.49%^b^	-	10.34
	early RM (3) PGT-A	-	58.75%	-	-	47.77%,60.98%^b^	-	14.13
	early RM (4) PGT-A	-	56.25%	-	-	34.18%,48.21%^b^	-	31.11
	LM (≥1) PGT-A	-	83.33%	-	-	61.11%,68.75%^b^	-	13.33
Liu et al. (27)	iRPL aged<35 PGT-A	-	52.30%	54.80%	-	-	4.80%	26.10%
	iRPL aged >35 PGT-A	-	47.90%	46.80%	-	-	4.30%	22.70%
	aged<35 PGT-M	-	62.90%	62.90%	-	-	3.10%	3.10%
	aged>35 PGT-M	-	66.70%	66.70%	-	-	16.70%	14.30%
Mantravadi et al. (25)	RPL PGT	-	-	-	-	32.3%	-	-
	RPL NO PGT	-	-	-	-	30%	-	-
Fodina et al. (28)	RIF PGT	-	-	49.3%	-	-	17.9%	4.5%
	RIF NO PGT	-	-	44.4%	-	-	5.6%	1.4%
Bhatt et al. (29)	RPL PGT-A	-	-	-	-	47.70%	9.90%	10.80%
	RPL	-	-	-	-	33.60%	11.50%	12.60%
Lei et al. (30)	RPL PGT-A	53.3%, 31.6%^b^	-	-	46.1%, 27.3%^b^, and 86.5%^c^	44.9%, 26.6%^b^, and 84.3%^c^	-	15.70%
	RPL	38.4%, 23.6%^b^	-	-	25.1%, 15.4%^b^, and 65.4%^c^	25.1%, 15.4%^b^, and 65.4%^c^	-	34.60%
Rao and Mantravadi (31)	RIF PGT-A	-	47%	-	-	-	-	-
	RIF	-	42%	-	-	-	-	-
Cozzolino et al. (32)	M-RIF >3	901	34.20%	-	35.89%	-	-	-
	M-RIF >3 PGT-A	58	38.20%	-	45.90%	-	48.9	-
	S-RIF > 5	206	34.80%	-	34.01%	-	35.5	-
	S-RIF > 5 PGT-A	29	39.80%	-	36.11%	-	0	-
Gu et al. (33)	RIF PGT-A	56.9%	-	49.5%	-	43.1%	-	-
	RIF NO PGT-A	33.9%	-	31.2%	-	25.7%	-	
Pantou et al. (34)	RM PGT	-	61%	-	-	50%	0	18.1%
	RM NO PGT	-	70%	-	-	12.5%	7.1%	75%
	RIF PGT	-	69.5%	-	-	47.8%	12.5%	18.75%
	RIF NO PGT	-	33.3%	-	-	19%	14.2%	21.4%
Pavlovic et al. (35)	RPL PGT	-	-	58.8%	-	44.3%	-	12.4%
	RPL NO PGT	-	-	45.6%	-	32.0%	-	12.2%
Du et al. (36)	RIF PGT-A	-	-	71.19%	55.93%	-	-	21.43%
	RIF NO PGT-A	-	-	56.30%	45.38%	-	-	19.40%

The superscript letter “a” refers to per patient, “b” refers to per ovarian stimulation/retrieval cycle, “c” refers to per pregnancy cycle, those without superscript letter refers to per embryo transfer, “-” refers to the missing data. PGT-A, Preimplantation genetic testing for aneuploidy; RRF, Recurrent reproductive failure; RIF, Recurrent implantation failure; RPL, Recurrent pregnancy loss; RM, Recurrent miscarriage; OD, Oocyte donors; RM, Repeated miscarriage; IR, Implantation rate; CPR, Clinical pregnancy rate; LBR, Live birth rate; OPR, Ongoing pregnancy rate; BPLR, Biological pregnancy loss rate; and MR, Miscarriage rate.

### 3.2 The aneuploidy rate increased in RRF, especially in patients with advanced age

Aneuploid embryos were commonly observed in the blastocysts from the IVF procedures. The euploidy rate was reported to be 56.4, 39.1, 42.8, and 25.5% in the excellent (≥3AA), good (3, 4, 5, 6 AB and BA), average (3, 4, 5, 6 BB, AC, and CA), and poor (≤3BB) blastocyst morphology groups, respectively (11). In RRF sufferers with blastocysts available for biopsy, the aneuploidy rate kept in line with maternal age. Sato et al. (20) reported that the aneuploidy rates were 43, 63, 69, and 91% in RPL sufferers and 56, 77, 77, and 94% in RIF sufferers according in the age groups of 35–36, 37-38, 39-40, and 41–42. Tong et al. (9) found that the aneuploidy rate in patients with RIF aged >38 years was significantly higher than that aged <38 years (68.9 vs. 39.9%, *p* < 0.001). Liu et al. (27) also reported that the aneuploidy rate in the idiopathic RPL group aged >35 years was higher than those aged <35 years (68.6 vs. 48.9%).

It should be noted that mosaic embryos were commonly observed according to the results of PGT-A based on CCS. Mosaicism is defined as the presence of ≥2 cell populations with different chromosomal constitutions within the same embryo (37), which may be contributed by many factors such as mitotic errors, amplification bias, contamination and the PGT-A provider, etc. (38). It was reported that the low-range mosaic embryos (<50%) showed a higher ongoing pregnancy rate and lower miscarriage rate, while a high-range mosaicism detection (>50%) was associated with whole chromosome aneuploidy in a significant proportion of cases. Therefore, cutoff 50% of mosaicism was recommended as a reference in clinical management (39). According to previous literature, the rate of mosaic embryo was 9.3 and 5.2% in RIF sufferers aged <38 and > 38 years (9), while 9.1 and 5.3% in RPL sufferers aged <35 aged group and RPL aged >35 aged group, respectively (27). This suggested that mosaicism rate did not always consistent with maternal age.

### 3.3 PGT-A could optimize the reproductive outcomes in the general population with RRF

#### 3.3.1 Patients using PGT-A had better reproductive outcomes than those not using PGT-A in RRF

In 2019, Kim et al. (21) retrospectively reviewed the reproductive outcomes of RPL sufferers undergoing their first single embryo transfer (ET). Results found that RPL sufferers using PGT-A (*n* = 660) had significantly higher CPR (73 vs. 61%, *p* = 0.01), LBR (62 vs. 41%, *p* < 0.01), and reduced clinical pregnancy loss rate (15 vs. 32%, *p* < 0.01), compared with RPL sufferers not using PGT-A (*n* = 101). In the same year, Lei et al. (30) reported that RPL sufferers using PGT-A (*n* = 212) had acquired higher LBRs per cycle start (26.6 vs. 15.4%, *p* = 0.0004) and transfer (44.9 vs. 25.1%, *p* < 0.0001), and lower MR (15.7 vs. 34.6%, *p* = 0.0007) than those not using PGT-A (*n* = 294). In 2020, retrospective study of Mantravadi et al. (25) reported that the MR (9.68 vs. 23.33%, *p* = 0.0610) was lower in patients with idiopathic RPL using PGT-A (*n* = 82) than those not using PGT-A (*n* = 30), although the LBR and take home baby rates were not significantly different. Also in 2020, Sato et al. (20) made a multi-center prospective study which revealed that no significant differences were observed in the LBR and the MR in RPL sufferers given or not given PGT-A. However, PGT-A improved the LBR per embryo transfer in the RPL (52.4 vs. 21.6%, *p* = 0.028; PGT-A group vs. non-PGT-A group = 41 vs. 38). Additionally, PGT-A reduced BPLR in the RPL group (12.5 vs. 45.0%, *p* = 0.03). Similarly, Sui et al. (24) conducted a prospective randomized clinical trial (study group vs. control group = 104 vs. 103), results also revealed that PGT-A significantly increased the OPR (55.34 vs. 29.81%, *p* < 0.05), LBR (48.54 vs 27.88%, *p* < 0.05), and decreased the MR (0 vs 14.42%, *p* < 0.05) on a per-patient analysis in RPL sufferers. In 2021, Bhatt et al. (29) conducted a retrospective study which included IVF-FET cycles from 2010 to 2016 in the Society of Assisted Reproductive Technologies Clinical Outcomes Reporting System. The results revealed that PGT-A increased the LBR (48 vs. 34%, *p* < 0.001) and CPR (59 vs 47%, *p* < 0.001) and decreased the BPLR (9.9 vs 11.5%, *p* = 0.02) and MR (11 vs. 13%, *p* = 0.02) in RPL sufferers (PGT-A vs. non-PGT-A = 3,241 vs. 3,351). In 2022, Pantou et al.’s (34) retrospective study revealed that in the RM group, a significant decrease of early pregnancy loss rate (18.1 vs. 75%, *p* = 0.001) and significant increase in LBRs per transfer (50 vs. 12.5%, *p* = 0.002)/per patient (36 vs. 12.5%, *p* = 0.026) were observed in the PGT-A group compared with the non-PGT-A group (*n* = 25 vs. 40). In 2023, Pavlovic et al. (35) retrospectively compared the reproductive outcomes of idiopathic RPL sufferers between the PGT-A group and the non-PGT-A group. The use of PGT-A tested embryos resulted in significant increase in CPR (58.8 vs. 45.6%, *p* = 0.007) and LBR (44.3 vs. 32.0%, *p* = 0.011) compared to cycles using untested embryos. After adjusting for confounding factors, LBR remained significantly increased in the PGT-A cycles compared with the non-PGT-A cycles (OR = 2.26, 95%CI 1.19–4.31). However, the use of an euploid embryo does not significantly decrease MR (12.4 vs. 12.2%, *p* = 1.000).

For RIF, in Sato’s above mentioned study (20), the LBR per ET were significantly increased in RIF sufferers with PGT-A than those without PGT-A (62.5 vs. 31.7%, *p* = 0.016, PGT-A group vs. non-PGT-A group = 24 vs. 42). Additionally, BPLR was significantly reduced in the RIF PGT-A group compared with RIF NO PGT-A group (10.5 vs. 40.9%, *p* = 0.04). Fodina et al.’s (28) retrospective study reported that PGT-A group showed statistically significant higher chance in achieving both biochemical (17.9 vs. 5.6%, *p* = 0 0.01) and clinical pregnancy (49.3 vs. 44.4%, *p* = 0.049), as compared to those who did not undergo PGT-A (*n* = 72 vs. 22) in RIF sufferers. In the same year, Rao’s (31) retrospective study also reported that IR was higher in the PGT-A group (*n* = 54) than the control group (*n* = 189) (47 vs. 42%), although the difference was not significant. In 2022, Gu et al. (33) conducted a retrospective analysis which revealed that the positive serum human chorionic gonadotropin (56.9 vs. 33.9%, *p* < 0.01), clinical pregnancy (49.5 vs. 31.2%, *p* < 0.01), live birth (43.1 vs. 25.7%, *p* < 0.01), and fetal heart rates (50.0 vs. 29.8%, *p* < 0.01) per transfer were significantly higher in the RIF-PGT-A group (*n* = 209) than the RIF-non-PGT-A group (*n* = 257). In Pantou’s (34) above mentioned study, a significant increase in the IR (69.5 vs. 33.3%, *p* = 0.005) and the LBR per transfer (47.8 vs. 19%, *p* = 0.015) was observed between PGT-A and non-PGT-A group in RIF sufferers (*n* = 30 vs. 42). These studies demonstrated that PGT-A could optimize the reproductive outcomes in RRF than those not using PGT-A.

#### 3.3.2 Patients with RRF using PGT-A had no inferior reproductive outcomes than patients without RRF history

In 2019, Kim’s (21) above mentioned study revealed that the Cin patients with RPL CPR using PGT-A (*n* = 660) was comparable (73 vs. 72%, *p* = 0.01) to that of infertile patients using PGT-A without RPL history (*n* = 3,975), although the clinical pregnancy loss rate was higher (15 vs. 12%, *p* < 0.01). In the same year, Wang et al. (23) conducted a retrospective cohort study that enrolled patients who had their first IVF cycle with PGT-A. It revealed that the positive β-HCG (65.9 vs. 68.9%), ongoing pregnancy (43.9 vs. 53.6%), and total pregnancy loss rates (33.3 vs. 21.4%) did not significantly differ in patients with RPL (*n* = 41) compared with patients without a history of miscarriage (*n* = 183). Bhatt et al.’s (29) above mentioned study also revealed no difference was observed in the reproductive outcomes between patients with RPL using PGT-A and those with tubal factors. In 2020, Lee et al. (22) retrospectively compared the reproductive outcomes in patients who experienced RIF (*n* = 82), RM (*n* = 82), and oocyte donors (OD) (*n* = 45) using PGT-A. Results showed that the LBR were similar among patients RIF, RM, and OD groups (51.6 vs. 55.9 vs. 57.1%). These studies suggested that patients with RRF using PGT-A had comparable reproductive outcomes than patients without RRF history.

However, study of Murugappan et al. (19) concluded that PGT-A could not improve the reproductive outcomes of patients with RRF. This retrospective study, which included 112 RPL patients desired who preimplantation genetic screening and 188 patients who chose expectant management (without further examination and treatment), revealed that the rates of CPR, LBR, and MR were similar between the PGT-A and expectant management groups. Moreover, the median time to pregnancy was even longer in the PGT-A group than in the expectant management group (6.5 vs. 3.0 months). However, it should be noted that in this paper patients with expectant management were used as a control, while the control group usually refers to RRF sufferers who do not use PGT-A or patients without RRF history who underwent PGT-A in other papers (40).

### 3.4 PGT-A could improve the reproductive outcomes in all age groups of RRF, especially in the advanced age group

In 2014, Greco et al. (18) conducted a retrospective study to investigate the effects of PGT-A in patients with RIF aged <36 years. The results revealed that the IR (68.3 vs. 22%, *p* = 0.001) and CPR (68.3 vs. 21.2%, *p* = 0.001) were significantly increased in the RIF PGT-A group (*n* = 43) than RIF NO PGT-A group (*n* = 33). On the other hand, the RIF PGT-A group had similar IR (68.3 vs. 70.5%, *p* = 1) and CPR (68.3 vs. 70.5%, *p* = 1), compared with NO RIF PGT-A group. Du et al. (36) also reported that the CPR was significantly increased in RIF PGT-A group (*n* = 59) compared with RIF NO PGT-A group (*n* = 119) (71.19 vs. 56.30%, *p* = 0.039) in RIF patients aged <38 years old. The OPR was also higher in the PGT-A group than the RIF without PGT-A group (55.93 vs. 45.38% *p* = 0.214), although the difference was not significant. These results indicated that PGT-A could optimize the reproductive outcomes in patents <38 years old.

Keiichi Kato (41) conducted a retrospective study which enrolled 32 patients who underwent PGT-A (18 in the RIF protocol and 14 in the RPL protocol) and 2,556 patients with IVF treatment at the same period for women aged 35–42 years in 2023. Results revealed that RPL patients with PGT-A had acquired increased LBR per ET (80.0 vs. 0, *p* = 0.005) and reduced MR (20.0 vs. 100.0%, *p* = 0.0098), compared with RPL sufferers without PGT-A. In the RIF sufferers, the PGT-A group also had better reproductive outcomes with higher LBR per ET [90.0 vs. 69.2% (*p* = 0.2313)], and lower MR (0 vs. 10.0%, *p =* 0.3297), although the difference was not significant. In 2021, Tong et al. (17) retrospectively compared the reproductive outcomes in RIF patients who underwent PGT-A between the younger (<38 years) and advanced (>38 years) age groups. Results revealed that there were no significant differences in the IR (39.1 vs. 51.0%), CPR (39.1 vs. 48.0%), and MR (4.3 vs. 7.8%) per ET between the two groups. In the same year, the above mentioned study conducted by Bhatt (30) revealed that in RPL sufferers, the adjusted odds ratio comparing IVF-FET with PGT-A vs. without PGT-A for live birth outcome was 1.31 (95% CI: 1.12, 1.52) for age < 35 years, 1.45 (95% CI: 1.21, 1.75) for ages 35–37 years, 1.89 (95% CI: 1.56, 2.29) for ages 38–40, 2.62 (95% CI: 1.94–3.53) for ages 41–42, and 3.80 (95% CI: 2.52, 5.72) for ages >42 years. These studies implied that PGT-A was beneficial in both young and patients with advanced age, particularly in patients with advanced age.

### 3.5 Effectiveness of PGT-A in RRF became limited with increased pregnancy failure times

In 2020, Sui et al.’s (24) above mentioned study revealed that the benefits of PGT-A were limited in patients with >2 failed PGT-A cycles (who failed to achieve ongoing pregnancy). In the same year, another retrospective multi-center cohort study by Cozzolino et al. (32) also reported that PGT-A could significantly improve the IR and OPR in the moderate RIF group (>3 implantation failures). However, the IR and OPR were not different in the severe RIF group (>5 implantation failures). Similarly, Ni et al.’s (26) retrospective study concluded that compared with the control group (patients using PGT-A after one spontaneous abortion with abnormal genetic testing results in aborted villus tissues and women with ≤2 IFs and ≤ 1 BPL, *n* = 103), patients with ≥4 previous early miscarriages (*n* = 56) had a significantly increased early miscarriage rate (6.58 vs. 31.11%, *p* < 0.001) and a decreased live birth rate (53.49 vs. 34.18%, *p* = 0.007) after euploid transfer. These studies demonstrated that PGT-A’s effectiveness was limited in patients with multiple pregnancy losses.

## 4 Discussion

Overall, our results indicated that PGT-A based on blastocyst biopsy and CCS procedures could optimize the reproductive outcomes of patients with RRF, which could be expected as the incidence of chromosomal abnormalities was higher in these patients. However, it should be noted that PGT-A cannot identify all possible genetic abnormalities or developmental defects. It could not guarantee successful pregnancy, which requires embryo with good quality and endometrial receptivity (42). As known, RPL and RIF are both complex and multifactorial condition. It is critical that RPL or RIF sufferers be properly evaluated to identify all possible causes and treated individually. Furthermore, they should be recommended to have prenatal diagnosis during the pregnancy period (27, 35). Their offspring should also have postnatal follow-up (25). In the future, the reproductive outcomes of RRF may be furtherly improved by artificial intelligence which could play a role in the following aspects: ultrasound monitoring of folliculogenesis, endometrial receptivity, embryo selection based on quality and viability, and prediction of post implantation embryo development, etc. (42).

In sub-analysis, we found that PGT-A was effective in RRF patients of any age, especially in patients with advanced aged. This made sense as the aneuploidy rate increased in patients with advanced maternal age in PGT-A cycles (16, 17, 20). However, it should be noticed that chances of women with advanced age getting blastocysts are less as their ovarian reserve is decreased. Deng et al. (43) reported that in patients with poor ovarian response, PGT-A cycles had less chance to reach embryo transfer compared with those not using PGT-A (13.7 vs. 70.6%, *p* < 0.001), and no difference were observed in the LBR per oocyte retrieval in cycles using or not using PGT-A (6.6 vs. 5.4%, *p* = 0.814). 31 PGT-A cycles were needed to avoid one clinical miscarriage. Therefore, PGT-A should be cautiously used for the population with advanced maternal age with poor ovarian response. Also, our results revealed that >3 instances of previous RRF or > 2 cycles of PGT-A cycles limited the benefits of PGT-A. This could be explained by the fact that no difference was observed in the prevalence of chromosomal abnormalities in couples with 2 and ≥ 3 pregnancy losses (44, 45), and PGT-A only selected euploidy embryos instead of changing the embryo pool (46). On the other hand, some other factors also affected the successful pregnancy rate other than an aneuploid embryo, such as thrombophilia, immunology, metabolic/endocrinological abnormalities, and anatomical abnormalities (47). Therefore, the benefits of PGT-A were not obvious in RRF with multiple pregnancy losses.

The primary limitation of this review was the paucity of high-quality studies which only included two prospective studies. Second, most studies did not show concomitant factors, such as AMH, previous times of pregnancy loss and other endocrine and immune disorders. As already known, AMH is an independent variable of increased aneuploidy embryo rate (27, 48). And previous occurrences of pregnancy loss and other endocrine and immune disorders were also closely associated with reproductive outcomes during the PGT-A cycles. Therefore, a direct comparison was challenging, because of the heterogeneity in patient cohorts of these studies.

## 5 Conclusion

Overall, PGT-A was beneficial for patients with RRF, especially in advanced aged patients. However, in patients with decreased ovarian reserve, the benefits of PGT-A may not be obvious as the probability of getting an euploid embryo was lower. In addition, PGT-A may have limited benefits for patients with multiple occurrences of pregnancy loss.

## Data availability statement

The original contributions presented in the study are included in the article/supplementary material, further inquiries can be directed to the corresponding author.

## Author contributions

YM drafted the manuscript and participated in data collection and analysis. YL participated in the design of the study and performed the statistical analysis. YC participated in its design and coordination. JZ, XK, and XL participated in data collection and analysis. FW participated in the design of the study and revised the manuscript. All authors contributed to the article and approved the submitted version.

## Glossary

PGT-A: Preimplantation genetic testing for aneuploidy
RRF: Recurrent reproductive failure
RIF: Recurrent implantation failure
RPL: Recurrent pregnancy loss
RM: Recurrent miscarriage
IVF: *In vitro* fertilization
ET: Embryo transfer
FISH: Fluorescence *in situ* hybridization
CCS: Comprehensive chromosomal screening
aCGH: Array comparative genomic hybridization
qRT-PCR: Quantitative real-time PCR
NGS: Next-generation sequencing
EM: Expectant management
OD: Oocyte donors
RM: Repeated miscarriage
IR: Implantation rate
CPR: Clinical pregnancy rate
LBR: Live birth rate
OPR: Ongoing pregnancy rate
BPLR: Biological pregnancy loss rate
MR: Miscarriage rate
